# DOES AGEISM ACCELERATE BIOLOGICAL AGING

**DOI:** 10.1093/geroni/igac059.1849

**Published:** 2022-12-20

**Authors:** Mingxin Liu, Alan Cohen, Tamas Fulop, Véronique Legault, Carine Betrisey, Melanie Levasseur

**Affiliations:** Université de Sherbrooke, Sherbrooke, Quebec, Canada; Université de Sherbrooke, Sherbrooke, Quebec, Canada; Université de Sherbrooke, Sherbrooke, Quebec, Canada; Université de Sherbrooke, Sherbrooke, Quebec, Canada; Université de Sherbrooke, Sherbrooke, Quebec, Canada; Université de Sherbrooke, Sherbrooke, Quebec, Canada

## Abstract

Defined as, “stereotype, prejudice, and discrimination directly towards people because of their age”, ageism may contribute to adverse health outcomes, accelerate aging process, and increase the burden on health and social services. Little is known about the ageism impact on biological aging. Secondary analysis of the American Health and Retirement Study (2012 and 2016 waves) was carried out. Participants were asked: the self-perception of aging (SPA), the causes of receiving discrimination, including ageism as one of the causes, and the frequency of receiving such discrimination. The aging rate was measured using two distinct measurements: homeostatic dysregulation (using Mahalanobis distance on 44 biomarkers, n= 9934, 2016 wave) and epigenetic aging clocks (n=4018, 2016 wave). The influence of perceived ageism (current or previous waves) on the aging rate was modelled with linear models using biological aging (aka. homeostatic dysregulation and epigenetic age) as the dependent variable (outcome), ageism as the exposure, with considering confounders: sex, depressive symptom. The results show that more negative SPA, either from the previous (2012) or the same wave (2016), is associated with elevated homeostatic dysregulation (e.g. the slope increases from 1.20 to 1.34, p< 0.001, previous wave) and increasing epigenetic age (e.g. DNAm PhenoAge, the slope increases from 53.81 to 61.14, p< 0.001, current wave). The association between the ageism receiving frequency and biological aging is similar but less significant. The results demonstrate that ageism is associated with accelerated biological aging. More interventions are called to combat ageism and foster the health and wellbeing of the older adults.

